# Sensory modulation in preterm children: Theoretical perspective and systematic review

**DOI:** 10.1371/journal.pone.0170828

**Published:** 2017-02-09

**Authors:** Tinka Bröring, Kim J. Oostrom, Harrie N. Lafeber, Elise P. Jansma, Jaap Oosterlaan

**Affiliations:** 1 Department of Medical Psychology, VU University Medical Center, Amsterdam, The Netherlands; 2 Department of Neonatology, VU University Medical Center, Amsterdam, The Netherlands; 3 Department of Epidemiology and Biostatistics, EMGO+ Institute for Health and Care Research and Medical Library, VU University Medical Center, Amsterdam, Netherlands; 4 Department of Clinical Neuropsychology, VU University Amsterdam, Amsterdam, The Netherlands; Vanderbilt University, UNITED STATES

## Abstract

**Background:**

Neurodevelopmental sequelae in preterm born children are generally considered to result from cerebral white matter damage and noxious effects of environmental factors in the neonatal intensive care unit (NICU). Cerebral white matter damage is associated with sensory processing problems in terms of registration, integration and modulation. However, research into sensory processing problems and, in particular, sensory modulation problems, is scarce in preterm children.

**Aim:**

This review aims to integrate available evidence on sensory modulation problems in preterm infants and children (<37 weeks of gestation) and their association with neurocognitive and behavioral problems.

**Method:**

Relevant studies were extracted from PubMed, EMBASE.com and PsycINFO following Preferred Reporting Items for Systematic Reviews and Meta-Analyses (PRISMA) guidelines. Selection criteria included assessment of sensory modulation in preterm born children (<37 weeks of gestation) or with prematurity as a risk factor.

**Results:**

Eighteen studies were included. Results of this review support the presence of sensory modulation problems in preterm children. Although prematurity may distort various aspects of sensory modulation, the nature and severity of sensory modulation problems differ widely between studies.

**Conclusions:**

Sensory modulation problems may play a key role in understanding neurocognitive and behavioral sequelae in preterm children. Some support is found for a dose-response relationship between both white matter brain injury and length of NICU stay and sensory modulation problems.

## Introduction

Advances in perinatal and neonatal intensive care have led to markedly increased survival rates in premature infants. Unfortunately, this reduced mortality is accompanied by an increased morbidity and high prevalence of neurodevelopmental problems, including neurocognitive and motor sequelae [1–5]. In addition, behavioral impairments in terms of increased incidence of both attention deficit hyperactivity disorder (ADHD) and autism spectrum disorders (ASD) are found in preterm children [6–9]. At school age, preterm born children have a two to threefold risk to develop ADHD and ASD [10,11]. Moreover, all these sequelae may translate in school difficulties, such as grade repetition, lower academic achievement levels and extensive use of special educational services [12–14]. Our current understanding of the mechanisms underlying the neurodevelopmental impairments in preterm children is still incomplete. This review aims to elucidate these impairments in terms of sensory processing problems and, specifically, sensory modulation problems.

Neurodevelopmental sequelae in preterm children are generally considered to result from early brain damage due to hypoxia-ischemia and inflammation [15], typically caused by concomitant medical conditions, such as bronchopulmonary dysplasia (BPD), necrotizing enterocolitis (NEC) and sepsis [16–19]. Premature infants tend to develop cerebral hypoxia-ischemia, especially in white matter, because of anatomical and physiological vulnerabilities of the vascular system. Furthermore, inflammation is common in preterms due to maternal intra-uterine infection and postnatal sepsis because of the immature immune system and is hypothesized to lead to inflammatory responses with subsequently raised levels of blood cytokines [15]. Some of the cytokines are toxic to oligodendrocyte progenitors (pre-OLs), disrupting the maturation of myelin-forming oligodendrocytes[15,20]. In addition to this cytokine injury, both hypoxia-ischemia and inflammation can lead to further damage to pre-OLs by the mechanisms of excitotoxicity and enhanced apoptosis caused by free radical attack, in turn exacerbating diffuse white matter damage and leading to periventricular leucomalacia (PVL) [15,21]. Volpe described hypoxia-ischemia and inflammation as two mutually potentiating pathogenetic mechanisms for developing ‘encephalopathy of prematurity’, which is a constellation of PVL and associated neuronal/axonal disease [15,21,22]. This neuronal/axonal disease is delineated by decreased volumes of the cerebral white matter, thalamus, basal ganglia, cerebral cortex, brainstem, and cerebellum [15,23–25].

In addition to the mechanisms of hypoxia-ischemia and inflammation causing PVL and axonal/neuronal disease, environmental factors of the neonatal intensive care unit (NICU) further compromise normal brain development [20,26–29]. The NICU is a stressful environment to which the preterm infant’s rapidly developing but immature brain is particularly vulnerable. Animal models demonstrate that the brain has critical periods in development which require optimal environmental exposure to enhance brain development [27,30]. Moreover, structural organization of the brain is altered by longer extra-uterine exposure as a consequence of the preterm birth, even without concomitant brain injuries [31–33]. In preterm infants, brain development may be further compromised by sensory overstimulation by bright lights, noise, nursery handling and repetitive pain in terms of inflammatory pain and NICU care procedures such as heel lancing, venipunctures and nasal suctioning [31,34,35]. In fact, preterms show structurally elevated stress markers such as increased heart rate and decreased oxygen saturation [36]. It is hypothesized that sensory overstimulation and repetitive pain propel excessive activation of central afferent pain pathways with subsequent excessive N-methyl-*D-*aspartate (NMDA) receptor activation resulting in, again, excitotoxic damage [26]. Indeed, NICU stressors are associated with decreased brain size in frontal and parietal regions and altered brain microstructure and functional connectivity within the temporal lobes [37]. In addition, normal brain lateralization may even be compromised by unstructured extra-uterine auditory stimulation before 30 weeks of gestation [35].

Together with the detrimental effects of sensory overstimulation and repetitive pain, also parental separation and sensory understimulation in terms of tactile, vestibular and kinesthetic deprivation are hypothesized to further compromise normal brain development, as afferent activity is reduced with a subsequent lack of NMDA activity which in turn induces apoptosis [26].

All of these destructive processes occur in the context of already insufficient self-regulatory abilities of the preterm and at a time where the sensory system is shaped by the amount and type of sensory experiences [31,38]. To counteract these challenges ‘Developmental Care’ interventions have been developed. For example, effective analgesia, kangaroo care, fine-tuned sensory stimulation and the Newborn Individualized Developmental Care and Assessment Program (NIDCAP) [30,39] to support the infant’s active self-regulation are believed to mitigate the adverse environmental effects of NICU care on the brain [1,27,40–42]. Thus, preterm birth as well as NICU environment can compromise brain development, especially cerebral white matter integrity. Cerebral white matter integrity is crucial for information processing, in particular sensory processing, and reduced white matter integrity is associated with sensory processing dysfunctions [31,43]. In fact, Owen and colleagues [43] recently showed a biological substrate of reduced white matter microstructure in children with sensory processing dysfunctions. Both primary sensory cerebral tracts and connective pathways to multimodal sensory regions were found to be affected. Therefore, the widespread white (and grey) matter abnormalities in preterms and altered structural brain organization in combination with the sensory over- and understimulation in the NICU strongly suggest that preterms are at risk for sensory processing dysfunctions.

Sensory processing can be defined as a three-stage construct including registration, integration and modulation of sensory stimuli. Dysfunctions in sensory processing are identified as sensory processing disorder (SPD) and pertain to the different stages in the sensory process [44–47]. Sensory registration dysfunctions range from basal sensory deficits (impaired sense of hearing, vision, taste, touch and/or, smell) to sensory discrimination disorder (SDD), in which children have difficulty discriminating or interpreting qualities of sensory stimuli in one or more sensory modalities [44]. Sensory integration dysfunctions include sensory-based motor disorder (SBMD), in which children show disturbances in integration of vestibular, proprioceptive, and visual information, resulting in poor postural control (postural disorder) or poor coordination (dyspraxia) [44]. Sensory modulation dysfunctions are defined as Sensory Modulation Disorder (SMD), in which children show an impaired regulation of the intensity of responses to sensory stimuli, resulting in hyporesponsiveness and/or hyperresponsiveness with subsequent maladaptive emotional, attentional, and motor responses to sensory stimuli [44,46,48].

A recent review found that SPD frequently occurs in preterm children, with some evidence for SDD (auditory, visual and tactile system) and SBMD [49]. Mitchell and colleagues [49] concluded that SMD was most frequently found in preterms up to age three years, with sensory overresponsivity being the most prevalent category. Indeed, registration and integration of sensory information are known to be compromised in preterm infants. Evoked potential (EP) studies show anomalous results in preterm infants on registration of all sensory modalities [31], ranging from lower activation of somatosensory cortical neurons and decreased thermal sensitivity [50,51] to frequent occurrence of cerebral visual impairment [52,53], abnormal auditory brain stem conduction [54,55] or smaller auditory event-related potentials [56] and abnormal vestibular EPs [57]. Integration dysfunctions in terms of dyspraxia (SBMD) and visual-motor integration problems are also known to be highly frequent in preterms [4,49,58]. Remarkably, modulation of sensory information has only been scarcely studied in preterm children. However, there are several reasons to suspect sensory modulation problems in preterm children. First, the pattern of diffuse white matter damage and axonal/neuronal disease in basal ganglia, cerebral cortex, brainstem and cerebellum[15,23,24] shows striking parallels with the so-called excitation-inhibition-modulation loop of sensory processing described by Koziol et al. [59]. This loop is thought to be crucial for effective sensory modulation, where the cortex, basal ganglia and cerebellum select, gate and regulate sensory stimuli, respectively. Secondly, sensory modulation is part of the already vulnerable self-regulatory abilities of the preterm, further compromised by both sensory overstimulation and understimulation in the NICU [60]. Third, common behavioral dysfunctions in preterm children, i.e. ADHD and ASD in particular, are strongly associated with problems in sensory modulation [61–63]. Both over- and underresponsivity are found in multiple sensory areas in ADHD and ASD [62,64–66]. Sensory modulation problems may form at least a partial link between prematurity and ADHD/ASD symptoms.

The current systematic review examines all available studies on the prevalence and nature of sensory modulation problems in preterm infants and children. Furthermore, we aim to integrate available evidence on risk factors of prematurity in association with sensory modulation problems and to elucidate associations between sensory modulation and neurocognitive and behavioral problems in preterms.

## Method

### Literature search and selection criteria

Relevant studies were retrieved using a comprehensive systematic search employing the bibliographic databases PubMed, EMBASE.com and PsycINFO (via EBSCO). Search terms included controlled terms from MeSH in PubMed and EMtree in EMBASE, thesaurus terms in PsycINFO as well as free-text terms. Search terms expressing ‘preterm children’ were used in combination with search terms comprising ‘sensory processing’/‘sensory modulation’ and ‘questionnaire/rating scale/test’ (S1 File. Search terms and strategy). Reference lists of the included studies were hand-searched for additional relevant publications. This review included all empirical studies that met the following inclusion criteria: the study had to 1) report on preterm children born < 37 weeks of gestation, and 2) assess the construct of sensory processing (disorder) in terms of sensory modulation, and 3) use a measurement (test, questionnaire, rating scale) to evaluate sensory processing/sensory modulation, and/or 4) evaluate a diagnosis of sensory processing disorder/sensory modulation disorder, and 5) be published in an English language peer-reviewed journal. Full-text articles were excluded if 1) the study did not report on preterm children born < 37 weeks of gestation or did not describe prematurity as a risk factor, or 2) no measurement (test, questionnaire, rating scale) was used to evaluate the construct of sensory processing (disorder) in terms of sensory modulation, or 3) the study was not published in an English language peer-reviewed journal, or 4) the study was not an empirical study. No limits were set on the age of the participants. All relevant studies published up to 5 December 2016 were included (S2 File. PRISMA checklist).

### Assessment of study quality

Two authors (TB and KJO) independently assessed the quality of the included studies using the Newcastle–Ottawa Scale [67]. This scale rates the quality of observational studies in terms of the selection of subjects (four criteria, four points), comparability of study groups (one criterion, two points) and outcome assessment (three criteria, three points). Total rating scores may range from zero to nine points, where higher scores indicate higher study quality (see Table 1). Differences in assessment between authors were solved by consensus. Since five studies did not use a control group, but did use a norm-referenced group, the selection criterion ‘Definition of Controls’, option ‘no history of disease’ was scored positive if norm-referenced data were used in the statistical analyses. All 13 cross-sectional/cohort studies (and two RCT/intervention studies) were evaluated with the Newcastle-Ottawa Scale, but this scale does not allow the assessment of the three included population-based studies because of lack of comparability on assessment criteria.

**Table 1:** Study characteristics and key results of included studies.

		*Group characteristics*		*Study characteristics*				*Key results*							
				Design	Measures	Aim	QS	Sensory modulation		Perinatal risk factors			Behavioral/neurocognitive measures		
Study		*n/*GA/age							p		Stat	p		Stat	p
*Standardized test*															
	Wiener et al., 1996	*n* PT/NC	56/228	Cross sectional	TSFI BSID	Sensory modula-tion and neurodevelopment in late preterm infants	6	Sensory modulation PT<NC:		-			No significant associations:		
		GA (wks)	<36					TSFI Total scale	< .01				TSFI-BSID		
		GA M(SD)	31(-)					all TSFI subscales	< .05						
		Age (mo)	7-18												
	Chorna et al., 2014	N PT/NC	72/-	Cross sectional	TSFI BSID-III	Sensory reactivity and neurodevelopment in preterms	4	Sensory modulation PT<norm-referenced group:		One week ↓GA ↑odds of lower tactile deep pressure score	1.68^e^	<.001	No significant associations:		
		BW (g)	<1500					abnormal score ≥ 1 TSFI subscale =82%:		Ocular-motor control-severe WMI	16.7^e^	<.001	Total number of deficient TSFI subscales-BSID-III		
		GA median(IQR)	28 (27-30)					Adaptive motor function= 40%							
		Age (mo)	4-12					Reactivity to tactile deep pressure=49%							
								Visual-tactile integration = 21%							
								Ocular–motor control =12%							
								Reactivity to vestibular stimulation = 21%							
	Cabral et al., 2016	*n* PT/NC	15/15	Cross sectional	TSFI	Sensory processing in preterms	6	Sensory modulation PT<NC:		-			-		
		GA (wks)	<37					TSFI Total scale	.01						
		GA M(SD)	31.3(1.8)					Reactivity to tactile deep pressure	<.001						
		Age (mo)	4-6 CA												
	Pekçetin et al., 2016	*n* PT/NC	34/34	Intervention study	TSFI	Efficiency of sensory integration interventions in preterms	6	Sensory modulation PT<NC (before intervention):		-			-		
		GA (wks)	<37					TSFI Total scale	<.001						
		GA M(SD)	-/-					all TSFI subscales	<.02						
		BW M(SD)	1503(482)												
		Age (mo)	7 CA												
*Standardized test and Caregiver Questionnaire*															
	Bart et al., 2011	*n* PT/NC	124/33	Cross sectional	TSFI ITSP	Sensory modula-tion and participation in preterms	7	Sensory modulation PT<NC:		Explained variance:			-		
		GA (wks)	34- 37					ITSP Oral	.04	SP+TSFI-GA	0.08^a^	<.001			
		GA M(SD)	34.9 (0.6)					ITSP Auditory	.03						
		Age (mo)	12					TSFI Total scale	.001						
								all TSFI subscales	< .01						
*Caregiver Questionnaire*															
	Case-smith et al., 1998	*n* PT/NC	45/22	Cross sectional	SRS BSID-II	Sensory respon-siveness and temperament in preterms	6	Sensory responsiveness PT<NC:		-			No significant associations:		
		GA (wks)	24-36					Total	.001				SRS-BSID-II		
		GA M(SD)	29.7(3.1)					Touch	.001						
		Age (mo)	12 CA										Positive associations within SRS:		
													Touch-difficult temperament	.63^b^	<.01
													Hearing-difficult temperament	.41^b^	<.01
													Vision-difficult temperament	.31^b^	<.05
	Janssen et al., 2009	*n* PT/NC	69/30	Cohort	ITSP BSID-II	Prevalence of psychopathology in preterms	6	Prevalence of psychopathology:		-			-		
		GA (wks)	25-36					PT(54%)>NC(30%):	<.05						
		GA M(SD)	2.7(2.4)					Multisystem develop-mental disorder: PT(6/69)>NC (0/30)							
		Age (mo)	12 CA					Regulatory disorder: PT (3/69)>NC(0/30)							
	Verkerk et al., 2011	*n* PT/NC	151/42	RCT	SP	Intervention study of sensory proces-sing in preterms	6	Sensory modulation PT=NC, except:PT+intervention>NC		-			-		
		GA (wks)	<32					Oral	.03						
		GA M(SD)	29.8(2.2)					PT+ care as usual< NC:							
		Age (mo)	44 CA					Endurance/Tone	<.001						
	Wickrema-singhe et al., 2013	*n* PT/NC	107/-	Cross sectional	ITSP/SP BSID-III WPPSI WISC	Sensory modula-tion in preterms	4	Sensory modulation PT<norm-referenced group:		No significant associations with perinatal factors			No significant associations:		
		GA (wks)	<32					Auditory	<.01				ITSP-BSID/WPPSI/WISC		
		GA M(SD)	28.3(2.3)					Tactile	<.02						
		Age (yrs)	1-8					Vestibular	<.01						
								All four quadrants	<.03						
								87%<-1SD any section/quadrant							
								58%<-1SD (>1section/quadrant)							
								39%<-2SD any section/quadrant							
								Low registration: 23%<-1SD							
								Other quadrants: 10/11% <-1SD							
	Eeles et al., 2013(a)	*n* PT/NC	253/65	Cohort	ITSP	Sensory modula-tion in relation to environmental and biological risk factors in preterms	7	Sensory modulation PT<NC:		Negative associations:			-		
		GA (wks)	<30					All sections	<.007	Auditory-WMA	-0.54^c^	.03			
		GA M(SD)	27.3(-)					All quadrants		Visual-WMA	-0.55^c^	.03			
		Age (yrs)	2 CA						<.002	Sensation avoiding-WMA	-0.58^c^	.003			
										Oral-NICU stay	-0.05^c^	.03			
										Vestibular-NICU stay	-0.07^c^	.02			
										Sensation seeking–NICU stay	-0.05^c^	.04			
	Eeles et al., 2013(b)	*n* PT/NC	241/-	Cohort	ITSP	Sensory modula-tion and neuro-development in preterms	4	See Eeles et al., 2013(a)		-			Positive associations:		
		GA (wks)	<30										Low registration-MDI	4.24^c^	.001
		GA M(SD)	27.3(-)										Auditory-MDI	4.33^c^	.001
		Age (yrs)	2 CA										Visual-MDI	3.81^c^	.01
													Touch-MDI	2.94^c^	.03
	Dudova et al., 2014	*n* PT/NC	75/-	Cross sectional	ITSP MCHAT CSBS-DP-ITC	Screening for autism spectrum disorders in preterms	4	Sensory modulation PT<norm-referenced group		No significant associations with perinatal factors			-		
		birth weight	<1500g					15% <-2SD (ITSP/MCHAT/CSBS-DP-ITC)							
		GA M(SD)	28.4(2.8)					42% <-2SD(≥1 questionnaires)							
		Age (yrs)	2 CA					12% ASD diagnosis confirmed by clinical assessment.							
	Rahkonen et al., 2015	*n* PT/NC	44/-	Cross sectional	ITSP BSID	Sensory modula-tion neonatal risk factors and neuro-development in extreme preterms	4	Sensory modulation PT<norm-referenced group		Sensation seeking PT<NC if:			-		
								52% <-1SD(≥1 quadrant/section):							
		GA (wks)	<28					Low registration: 23%		Grey+WMA	-	<.01			
		GA M(SD)	26.3(1.2)					Sensory avoiding: 18%		Surgical PDA	-	.01			
		Age (yrs)	2 CA					Sensation seeking: 14%		Oral PT<NC if:					
								Sensory sensitivity: 7%		Surgical PDA	-	<.01			
								Vestibular: 18%							
								Oral: 18%							
								Visual: 16%							
								Tactile: 9%							
								Auditory: 7%							
	Adams et al., 2015	*n* PT/NC	54/73	Cross sectional	SSP BRIEF-P EF tasks Vineland	Sensory modula-tion and executive/adaptive functioning	7	Sensory modulation PT(37%)<NC(12%):	.001	Explained variance in sensory modulation:			Negative associations Total SSP with BRIEF-P:		
		GA (wks)	<34					SSP Total	<.001	SSP Total -GA	0.16^a^	<.001	Total score	-.59^d^	<.01
		GA M(SD)	29.5(2.5)					Underresponsive/ seeks sensation	<.001	No other significant associations			Working memory	-.63^d^	<.01
		Age (yrs)	3-5					Movement	<.002				Inhibition	-.55^d^	<.01
								Auditory	<.001				PT+(elevated SSP) vs PT-(no elevated SSP):		
								Visual/auditory	<.001				EF battery-Gift wrap		.02
								Low energy/weak	.003				No significant associa-tion SSP-Vineland		
	Crozier et al., 2016	*n* PT/NC	160/-	Cohort	SSP	Prevalence and type of sensory processing differences	4	Sensory modulation problems in PT (<-1 SD)		PT+(SSP<-1SD) vs PT-(SSP>-1SD): negative association:			-		
		GA (wks)	<32					All domains: 46%		Apgar score	0.81^e^	.03			
		GA median (IQR)	26(25-28)					Underresponsive/ seeks sensation: 46%		Positive association					
		Age (yrs)	4.5					Movement:33%		NICU days	1.01^e^	.02			
								Auditory: 44%							
								Visual/auditory:46%							
								Tactile: 45%							
								Taste/smell: 31%							
								Low energy/weak: 40%							
								Underresponsive/ seeks sensation: 46%							
	May-Benson et al., 2009	*n* SPD/SPD+ ASD	1000/465	Population based	Clinical exam	Incidence peri-natal/develop-mental problems in children with SPD (and ASD)		No significant difference between SPD and SPD+ASD group on GA					-		
		% <37 wks SPD/SPD+ ASD	12.4/16					Prevalence of prematurity higher than national average in SPD+ASD group							
		Age (yrs)	3-17												
	Franci Crepeau-Hobson 2009	*n* NC	152	Population based	SSP	Perinatal risk fac-tors and sensory modulation		GA predicted SSP Total and subscales		Negative associations:			-		
		Age (yrs)	3-7							Total SSP-GA	-.16^b^	<.05			
										Tactile-GA	-.24^b^	<.05			
										Movemen -GA	-.20^b^	<.05			
										Underrespon-sive/seeks sensation-GA	-.14^b^	<.05			
										Explained variance:					
										Tactile-GA	.051^a^	.004			
	Van Hulle et al., 2012	*n* NC twins	978	Population based cohort	TBAQ	Sensory over-responsivity in typically developing twins		GA was associated with stability of sensory modulation problems		One week ↑GA ↓odds modulation problems both 2 and 7 years	.087^e^	.05	Stability of Tactile overresponsivity (2 and 7 years): positive association:		
		Age (yrs)	2 and 7							Stability of Tactile overresponsivity (2 and 7 years) negative association:			Object fear	4.1^f^	<.001
										GA	3.2^f^	.002	Social fear	2.8^f^	.006
													Soothability	2.1^f^	.04
													Stability of Auditory overresponsivity (2 and 7 years): positive association:		
													Social fear	2.2^f^	.03
													Soothability	2.1^f^	.04

*Note*. A dash (-) means that associations were not investigated.

^a^ = R^2^

^b^ = Pearson correlation

^c^ = Regression coefficient

^d^ = Spearman correlation

^e^ = odds ratio

^f^ = *t-*value.

Abbreviations: QS = quality score; PT = preterms; NC = normal controls; TSFI = Test of Sensory Functions in Infants; BSID (II/III) = Bailey Scales of Infant Development (II/III); ITSP = Infant/Toddler Sensory Profile; GA = gestational age; CA = corrected age; SRS = Sensory Responsiveness Scale; SP = Sensory Profile; RCT = randomized controlled trial; WPPSI = Wechsler Preschool and Primary Scale of Intelligence; WISC = Wechsler Intelligence Scales for Children; WMA = white matter abnormalities; NICU = neonatal intensive care unit; MDI = mental development index M-CHAT = Modified Checklist for Autism in Toddlers; CSBS-DP-ITC = Symbolic Behavior Scales Developmental Profile Infant-Toddler Checklist; ASD = autism spectrum disorder; SSP = Short Sensory Profile; BRIEF-P = Behavior Rating Inventory of Executive Function- Preschool Version; EF = executive functioning; IRQ = inter quartile range; SPD = sensory processing disorder; TBAQ = Toddler Behavioral Assessment Questionnaire (Sensory overresponsivity scale; similar to SSP).

### Definitions of prematurity

The World Health Organization (WHO) defines prematurity as birth before 37 weeks of gestation and subdivides prematurity in: moderate to late preterm birth (32–37 weeks), very preterm birth (28–32 weeks), and extremely preterm birth (< 28 weeks) [68].

### Operationalization of sensory modulation

The construct of sensory modulation is operationalized and measured differently between the studies. In this review, we use the framework developed by Dunn to organize the construct of sensory modulation [46–48,69]. The framework of Dunn can be conceptualized as a quadrant scheme with either high or low neurological perception thresholds on the rows, and either active or passive self-regulation on the columns. Using this quadrant scheme, four types of individuals can be distinguished: 1) individuals with high neurological perception thresholds and passive self-regulation strategies ('Low registration'); 2) individuals with high neurological perception thresholds and active self-regulation strategies ('Sensation seeking'); 3) individuals with low neurological perception thresholds and passive self-regulation strategies (‘Sensory sensitivity’), and 4) individuals with low neurological perception thresholds and active self-regulation strategies (‘Sensory avoiding’). Dunn used this framework to develop the widely used Sensory Profile, a rating scale that can be completed by caregivers [46,69–72].

### Measures

Across the 18 included studies two caregiver questionnaires (Sensory Profile [SP] [71]; Sensory Rating Scale [SRS] [73]) and one infant test battery (Test of Sensory Functions in Infants [TSFI]) [74] were used and described below (see also Table 1). A recent review on sensory processing measures shows that these three measures are reliable and valid measures of sensory processing (see for details a review by Eeles and colleagues (2013) [75]).

#### Sensory profile

The Sensory Profile (SP) is a caregiver-completed five-point scale questionnaire measuring sensory modulation abilities and problems in daily life [71]. Three versions exist: the 48-item Infant/Toddler Sensory Profile (ITSP) for ages birth-3 years [69], the 125-item standard SP for ages 3–10 years [71], and the Short Sensory Profile (SSP) for ages 3–10 years comprising 38-items. Both the ITSP and SP provide section and quadrant scores. The two rating scales comprise sections that pertain to five sensory systems, i.e. Auditory, Visual, Vestibular, Tactile and Oral systems, and a Multisensory section. Only the SP has eight additional sections, i.e. five modulation sections and three behavioral sections. The five modulation sections measure combinations of sensory input, concerning Endurance/tonus, Body position and movement, Movement in relation to activity level, Emotional responses and Visual input. The three behavioral sections describe Emotional/social reactions, Behavior and Perception thresholds for a response. Principal component analysis on the SP items has revealed nine factors. These factors pertain to the four quadrants of Dunn’s scheme [46], including Sensation seeking, Sensation avoiding/emotionally reactive, Sensory sensitivity and Low registration, and to five other factors, i.e. Low stamina/tonus, Oral-sensory sensitivity, Inattention/distractibility, Preference for sedentary activities and Fine motor/perceptual skills. Principal component analysis on items of the ITSP have only revealed the four quadrants. In the SSP seven factors are identified: Tactile sensitivity, Taste/smell sensitivity, Movement sensitivity, Auditory filtering, Low energy/weak, Underreactive/seeks stimulation and Visual/ auditory sensitivity.

Low scores on sections, factors and quadrants indicate sensory modulation problems and can be described as atypical (< -1 SD), as reflecting a probable difference (between -1 SD and -2 SD) and definite difference (<-2 SD). For the SP adequate reliability has been found for sections, quadrants and factors with Cronbach’s alpha scores of 0.63–0.91.[69,71] In the ITSP adequate reliability has been found, with Cronbach’s alpha scores of 0.70–0.86 for the quadrants, and 0.63–0.71 for the sections, with three exceptions for the Visual, Vestibular and Oral-sensory sections (0.44 < α < 0.55). Moreover, adequate test-retest reliability of ITSP has been found for sections (*r* = 0.86) and quadrants (*r =* 0.74). The SSP Total score has high reliability (α = 0.96) and discriminative validity, correctly identifying >95% children with and without sensory modulation dysfunction [70]. Internal consistency of SSP factors ranged from 0.70 to 0.90. Content and construct validity have been established for all versions [69,71].

#### Sensory rating scale

The Sensory Rating Scale (SRS) [73] is a caregiver-completed 136-item questionnaire to evaluate sensory modulation, referred to as sensory responsiveness, sensory defensiveness and temperament in infants aged 9–36 months. The SRS comprises six sections; Touch section, Movement/gravity section, Hearing section, Vision section, Taste/smell section, Temperament/general sensitivity section and a Total score. Adequate psychometric properties have been obtained: high to adequate reliability was found for the total scale, form a (α = 0.90) Total score (α = 0.83) and sections (0.65 < α < 0.82), with two exceptions for the Vision (α = 0.56) and Taste/Smell section (α = 0.46) [73]. Intra-rater reliability was high for mothers (r = 0.89) and fathers (r = .0.95), whereas inter-rater reliability was only moderate (r = 0.43). Content validity has been established. No research was conducted on construct and criterion-related validity [73].

#### Test of sensory functions in infants

The Test of Sensory Functions in Infants (TSFI) [74] is a 24-item test to assess sensory modulation (referred to as sensory processing and reactivity) in infants aged 4–18 months by presenting visual, tactile and vestibular stimuli to assess the intensity of the infant’s response. Scores can be calculated for five subscales, i.e. Response to tactile deep pressure, Visual-tactile integration, Adaptive motor, Ocular motor, and Reactivity to vestibular stimulation. The five subscales sum up to a Total scale. Scores can be categorized as normal, at risk, or deficient, using normative data derived from normal, delayed and regulatory disorder groups of infants or children. Adequate psychometric properties have been obtained for the TSFI: the test-retest reliability for the Total scale score was *r* = 0.81 and ranged from *r =* 0.64–0.96 for the subtests scores, with a single exception for Reactivity to vestibular stimulation (*r =* 0.26). Inter-observer reliability is high with convergence between the raters of 81–96% for all scales [74]. Content and construct validity have been established.

## Results

### Study selection

**Fig 1:**
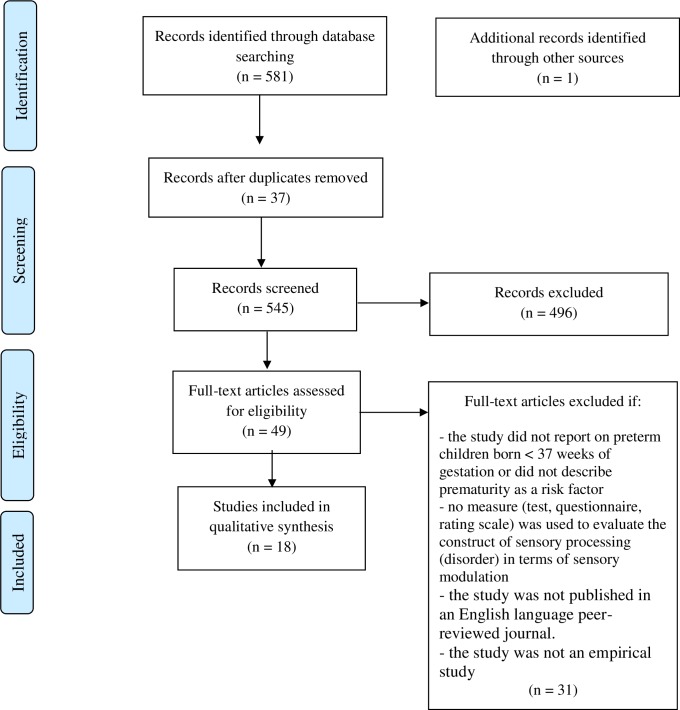
Flow chart of literature search and study selection. From: Moher D, Liberati A, Tetzlaff J, Altman DG. Preferred Reporting Items for Systematic Reviews and Meta-Analyses: The PRISMA Statement. PLoS Med 2009; 6: e1000097.

The literature search generated a total of 581 references. After removing duplicates of references that were selected from more than one database, 545 references remained. Title and abstracts were screened for relevance, by two authors (T.B. and K.O) independently, and 49 studies were further assessed for eligibility using the full text of the study report and the data extraction form (S3 File. Data extraction form). A total of 18 studies (published between 1996 and 2016) met the inclusion criteria and were included in the present review (Fig 1) [76].

### Study characteristics

The systematic literature search yielded 18 eligible studies of which 15 reported on sensory modulation in a preterm sample [77–91] and three reported on sensory modulation in general population samples [92–94], analyzing GA as a risk factor for sensory modulation problems (Table 1). Of the included studies, one study specifically reports on late preterm children [78]. Five studies report on the full spectrum of prematurity (22–37 weeks) [77,79,80,90,91]. Seven studies report on very preterm/very low birth weight (<1500g) children [81–84,86,87,89] and one study reports on extremely preterm children [85]. In the 15 studies in preterms, 22 groups of children were evaluated, including 1259 preterm and 542 controls. Nine studies were case-controlled [77–80,82,86,87,90,91]. Sample sizes of the preterms ranged from 15 to 253 and of the controls ranged from 15 to 228. Control populations in all the studies were matched with the preterms on one or more demographic feature (gender, age, number of siblings/multiple birth, socioeconomic status [SES]). Twelve studies [77–80,82–85,89–92] evaluated sensory modulation before or at two years corrected age (CA). Of the 15 studies reporting in a preterm sample; 33% are 1-year-olds or younger (CA), 30% are 2-year-olds (CA), 29% are 3–5 year olds and one sample, 8%, is cross-sectional (1–8 year olds). The TSFI was used in five of our included studies [77,78,89–91]. In 11 of our included studies a version of the SP was used; the ITSP and SP were combined in one study [81], the ITSP was used in six other studies [78,80,82–85]; the SP was used in one other studies [87], and the SSP was used in three studies [86,88,93]. The SRS was used in one of our included studies [79]. Eight studies contained data from the United States [77,79,81,86,89,92–94], five studies were conducted in Europe [80,84,85,87,91], two in Australia [82,83], one in Israel [78], one in Brazil [90] and another one in Canada [88].

### Sensory modulation

Evidence in support of sensory modulation problems in preterms [77–86,88–91] was reported in 14 preterm studies and two population-based studies reported significant associations between GA and sensory modulation problems [92,93]. The other two studies [87,94] did not find evidence for the idea that preterm birth is associated with sensory modulation problems.

#### Sensory profile

The ITSP/SP/SSP was used in 11 studies [78,80–88,93] of which ten [78,80–86,88,93] found that preterm born infants showed significantly more problems in sensory modulation compared to term-born controls or reference groups. Six studies reported explicitly on the SP/ITSP/SSP in preterms [78,81,82,85,86,88]. Problematic Auditory modulation was the most robust finding; all six studies found this section to be affected in preterms. All other sections (Visual, Vestibular, Tactile, Oral) were found in four out of the six studies. The Low registration quadrant was found to be (most) affected in five studies [81,82,85,86,88]. Three studies (also) found the other quadrants to be affected in preterms [81,82,85].

However, in all ten studies that found significantly more sensory modulation problems using the same measure, no clear pattern of problems emerged for the quadrants and/or sections, with the exception that three studies found Low registration (underresponsivity) to be the most affected quadrant with 23–46% of preterm children scoring <1 SD [81,85,88].

One cross-sectional study [81] found that the incidence of atypical score(s) on the ITSP/SP was similar across two different age groups with 37% of the 1–4 year olds (n = 70) and 43% of the 4–8 year olds (n = 37) obtaining at least one atypical score.

#### Sensory rating scale

One study used the SRS, showing that preterm born infants had more sensory modulation problems than term born children as assessed with the SRS Total score and most pronounced for Touch sensitivity [79].

#### Test of sensory functions in infants

Five studies used the TSFI [77,78,89–91]. Three studies found that preterm infants performed worse than term-born controls on the Total scale and all subscales, tapping into different aspects of sensory modulation (i.e. Response to tactile deep pressure, Visual-tactile integration, Adaptive motor, Ocular motor and Reactivity to vestibular stimulation) [77,78,91]. Cabral and colleagues [90] found that preterm infants performed worse on the Total scale and on their Response to tactile deep pressure in comparison to term-born controls. One study, with norm-referenced comparison, found that 82% of preterms had at least one at-risk/deficit range subscale score, with Response to tactile deep pressure and Reactivity to vestibular stimulation most frequently affected [89]. Also, Wiener et al. [77] found that with increasing age, preterms more frequently reached scores in the at-risk and deficit range. On Reactivity to vestibular stimulation, all preterms scored in the at-risk or deficit range, independent of their age.

### Sensory modulation and perinatal risk factors

Eight of the included studies investigated relations between prenatal, perinatal and neonatal factors, and sensory modulation problems [78,82,85,86,88,89,92,93]. Five studies found that GA was negatively associated with sensory modulation problems [78,86,89,92,93]. Three studies found that white (and grey) matter brain abnormalities were positively associated with sensory modulation problems (poor ocular motor control, auditory modulation, sensation seeking and sensation avoiding) [82,85,89], and two studies found that length of NICU stay was positively associated with sensory modulation problems (oral modulation and sensation seeking) [82,88]. In addition, Rahkonen et al. [85] found that surgical closure of patent ductus arteriosus (PDA) was positively associated with the Sensation Seeking quadrant and Oral modulation and Crozier et al. [88] reported that Apgar scores were associated with sensory modulation problems in very preterm children.

The population-based study of Franci Crepeau-Hobson [93] found that GA was negatively associated with the Total SSP score, Tactile sensitivity, Movement sensitivity and the Underresponsive/Seeks Sensation factor scores. Another population-based study, by Van Hulle et al. [92], showed that an increase of one week in gestational age decreased the odds of having sensory overresponsive symptoms at both 2 and 7 years of age, as measured with the Sensory Overresponsivity subscale (item content highly similar to SSP) of the Toddler Behavioral Assessment Questionnaire (TBAQ).[95] In addition, an interaction was found between GA and stability of tactile overresponsivity, such that the earlier a child was born, the more strongly early tactile symptoms were found to predict later tactile symptoms. Thus, symptoms of tactile overresponsivity were more stable across time among children born prematurely than among term-born children.

### Sensory modulation and neurocognitive functioning

The relationship between sensory modulation problems and cognitive development was examined in seven studies of which five found that sensory modulation problems were not significantly related to cognitive development [77,79,81,85,89]. However, two studies did find associations between sensory modulation problems and cognitive functioning [83,86]. Eeles et al. [83] found that lower scores in the Low registration quadrant and the Auditory, Visual and Touch sections were related to lower mental scores on the BSID (BSID II-III) [96]. Adams et al. [86] found that elevated numbers of sensory modulation symptoms (SSP Total score, Taste/smell sensitivity, Underresponsive/seeks sensation, Auditory filtering, Low energy/weak, Visual/auditory sensitivity) showed more executive impairment on the Behavior Rating Inventory of Executive Function- Preschool Version (BRIEF-P) Total score [97]. SSP total score had the highest correlation with the subscales Working memory and Inhibition. Also, the SSP total score was positively associated with inhibition/delayed gratification (Gift wrap) on a performance-based EF battery [86] when preterms were split in two groups (elevated SSP scores vs no elevated SSP scores).

### Sensory modulation and behavioral functioning

The relationship between sensory modulation and behavior was examined in five studies [79,80,84,92,94]. Both Dudova et al. [84] and May-Benson et al. [94] found evidence that sensory modulation problems and ASD coincide, by showing a higher prevalence of ASD and sensory modulation problems in preterm born infants than in term controls. Case-Smith [79] found moderate positive associations between both Hearing sensitivity and Vision sensitivity (SRS) and difficult temperament. Strong positive associations were found for Touch sensitivity, explaining 40% of the variance in temperament [79]. Janssens et al. [80] classified infants according to the Diagnostic Classification Zero to Three (DC:0–3) [98] with structured interviews, clinical observations, ITSP, BSID-II and a language inventory. The ITSP was used to diagnose Regulatory Disorders (RD) and Multisystem Developmental Disorder (MSDD). It was found that significantly more preterms (54%) than controls (30%) suffered from psychopathology. The most common diagnosed disorders in preterms were MSDD and RD, whereas none of the controls had MSDD or RD. Van Hulle et al. [92] found that sensory overresponsivity was associated with temperament dimensions of fear and soothability and that stability of sensory overresponsive symptoms over time was partly determined by fearful and less soothable temperaments. However, this was true for the complete sample of typically developing twins, and not specific for preterm born children.

### Risk of bias

Some selection bias is present in the 15 studies in preterm children due to both recruitment procedures and differences in terms of inclusion and exclusion criteria for preterms and controls. In only two out of 15 studies, a consecutive sample was included [84,85]. In all other studies, a fixed timeframe of inclusion was used or children shared a uniform selection method at one hospital or clinic, mitigating this effect of bias. Selection bias in the control groups mainly comprises the (absence of reports on) non-response rates due to convenience sampling [77–80,82,83,86,88,90,91]. However, all control groups were community controls and there were no preterm born children included in control groups. Exclusion criteria were not reported in five out of 15 studies [78,81,85,87,88]. Exclusion criteria (e.g. congenital/metabolic disease, major neurosensory disabilities, CP, language) were sufficiently described and equal in the remaining ten studies [77,79,80,82–84,86,89–91].

Preterms participating in the included studies differed in terms of baseline characteristics, including GA, birth weight, neonatal complications and social background characteristics. Of the 1259 included preterm infants, 10% were late preterm, 17% were born between 23–37 weeks, 70% were born very preterm and 3% were born extremely preterm. Yet, comparability between the preterm groups and the control groups is relatively high as almost all case-controlled studies matched on age, and four studies also matched on social economic status and/or gender [80,83,86,87].

Some performance bias advances as administration of the TSFI and cognitive tasks was not blinded [77–80,83,86,87,89–91]. However, most studies used the SP/ITSP which are based on parental reports and therefore not susceptible to performance bias [78,80–87]. Detection bias is present due to differences between the studies in terms of the measures used to assess sensory modulation, however, 11 out of 18 studies shared the same measurement (ITSP/SP/SSP). Attrition bias is common in observational studies with preterm children, but in almost half of the studies reasons for attrition were fully reported (death, refusal, language, emigration) and unlikely to confound results [78,80,84,85,87]. Reporting bias is low in all the included studies. Publication bias is a possible risk. This line of research in preterm children is relatively new and the topic is scarcely studied. It is possible that studies with non-significant results between preterm and term born children may not have been published. No conflicts of interest are reported in any of the studies.

## Discussion

The present study reviewed the empirical literature on sensory modulation problems in preterms. Evidence was found in support of sensory modulation problems in preterms [77–86,88–91]. It was found that prematurity may distort various aspects of sensory modulation, including problems across sensory modalities (auditory, visual, vestibular, tactile and taste) [81–83,85,86,88] and sensory modulation functions (Response to tactile deep pressure, Visual-tactile integration, Adaptive motor, Ocular motor and Reactivity to vestibular stimulation) [77,78,89–91] resulting in behavioral patterns of various nature (Low registration, Sensation seeking, Sensation avoiding/emotionally reactive, Sensory sensitivity) [81–83,85,86,88]. Consequently, the nature and severity of the sensory modulation problems differed widely between the studies. The observed heterogeneity in the distortions might be explained by differences between the studies in terms of the measures used to assess sensory modulation. Although even in the studies where the same measure (ITSP) was used, no clear pattern of problems emerged for either one of the quadrants and/or sections, with the exception that Low registration (underresponsivity) was the most affected quadrant [81,85,88]. A second explanation for the heterogeneity in the findings of the present review might be differences in the factors leading to sensory modulation problems in preterms, including altered cortical organization due to too early extra-uterine exposure [31], hypoxia-ischemia and inflammation leading to disturbances in cerebral white matter integrity, as well as under- and overstimulation during NICU stay due to parental separation and lights, noises, nursery handling and pain, respectively. Some preterms could have suffered more from overstimulation with excitotoxic damage and possible downregulation of the sensory system, while other preterms might have suffered more from understimulation with apoptosis and upregulation of the sensory system. Consequently, the atypical sensory modulation scores across the ITSP/SP quadrants are suggested to be an offshoot of originally adaptive responses to this down- and upregulation [82]. However, after the NICU stay, these regulatory responses may have become maladaptive, resulting in sensory modulation problems later in life [82]. The relatively high incidence of regulatory disorders among preterms would also fit this hypothesis [80].

The included studies that did not find sensory modulation problems [87,94] differed from the other studies in terms of their study design. Rather than using a comparative group design, Verkerk et al. [87] performed an intervention study within a sample of preterm born infants. Nevertheless, no significant differences in sensory modulation were found in this study in comparison with term born controls, except for Endurance/tone. May-Benson et al. [94] conducted an explorative descriptive study in children with ASD and SPD, in which prematurity was used as a dichotomous within-subject factor, whereas in the other population-based studies GA was used as a continuous variable, offering a statistically more powerful design to assess the effects of GA.

Our findings are in accordance with a recent review demonstrating greater risk of SPD in preterm born preschoolers [49]. Our review adds to that finding by showing that problems are not limited to preterm infants, but that sensory modulation problems are also evident in preterm children (1 to 8 years of age). Moreover, associations were described between sensory modulation and perinatal risk factors, neurocognitive and behavioral measures.

The mechanisms of brain development in preterms and the detrimental effects of NICU stay are highly suggestive for sensory modulation problems [7,43,51,59]. However, research on the etiological mechanisms causing sensory modulation problems in preterms is scarce. The current review has found relevant predictors for developing sensory modulation problems, including GA, birth weight, white (and grey) matter abnormalities, length of NICU stay and PDA [78,82,85,86,88,89,92,93]. These studies await replication, but the results suggest a dose-response relationship between both white matter brain injury and NICU stay and sensory modulation problems. However, given the correlational design of these studies, a causal relationship between sensory modulation problems and NICU environment and white brain matter abnormalities is not established.

The relationship between sensory modulation problems and cognitive development is still unclear. In the reviewed literature some study results suggest that sensory modulation problems are a separate and independent part of child development [77,79,81,85,89], whereas other studies found significant associations between sensory modulation and neurocognitive outcomes, including executive functioning problems [83,86]. These results suggest that children with low registration, described by high perception thresholds and passive self-regulation, are hampered in their learning opportunities due to little exploration and engagement. In addition, Adams et al. [86] found that sensory modulation problems coincided with problems in executive functioning, especially working memory and inhibition. These findings suggest that the vulnerable self-regulatory abilities of preterm infants in the NICU may grow into disrupted higher-order cognitive control in terms of executive functioning problems and sensory modulation problems later in life [99]. To increase our understanding of the possible relations between sensory modulation and cognitive development, more research is needed.

In addition, sensory modulation and behavior may be related [79,80,84,92,94]. Two included studies showed that sensory modulation problems and ASD coincide [84,94] and associations were found between sensory modulation problems and regulatory disorder and difficult, fearful and less soothable temperament [79,80,92]. These results are in accordance with studies in both ADHD and ASD, showing that problems in sensory modulation are strongly associated with ADHD and ASD symptoms [61–63]. Given the fact that ADHD and ASD symptoms are known to be elevated in preterms [6–9], a possible developmental trajectory emerges in which preterms with sensory modulation problem are at enhanced risk to develop symptoms of ADHD and ASD. In fact, sensory modulation problems may be one of the explanations for the high prevalence of ADHD and ASD symptoms found in preterms. This possible association between ADHD, ASD and sensory modulation also requires additional research.

Although the present review supports the idea that sensory modulation in preterm born infants is at stake, caution is required in interpreting the results due to risk of bias and limited quality of some studies. First, some selection bias is present in the studies due to recruitment procedures, different exclusion criteria and lack of comparability between groups of preterms. In addition, characteristics of the samples, if reported, vary in terms of neonatal complications, race and SES, hampering generalizability of findings. Second, common short-comings in research in preterm children, such as convenience sampling of control participants, high attrition rates, and sole use of norm-referenced data, are also present in some of the included studies. However, comparability between the preterm groups and the control groups is relatively high as almost all studies matched on age and social economic status and/or gender. Thirdly, the available studies on sensory modulation pertain to a restricted age group. Although three studies with different age ranges [81,86,88] show persistent sensory modulation problems in preterm children aged > 2 years and more apparent impaired sensory modulation with increasing age, this important finding awaits replication. Lastly, publication bias is a possible risk, as sensory modulation is a scarcely studied area in preterm children and studies with non-significant results may fail to be published.

Future research on sensory modulation in preterm children is clearly needed to replicate and extend the available results. Such studies need to be term-born controlled longitudinal studies combining sensory modulation measures with neurocognitive measures and behavioral measures tapping into ADHD and ASD.

## Supporting information

S1 File Search terms and strategy. (DOCX)

S2 File PRISMA checklist. (DOCX)

S3 File Data extraction form. (DOCX)
