# Supplementary material for: Sensory modulation in preterm children: Theoretical perspective and systematic review
Source: PLoS One. 2017 Feb 9;12(2):e0170828. doi: 10.1371/journal.pone.0170828 (PMC5300179; doi:10.1371/journal.pone.0170828)
Supplement: S1 File — (DOCX) [file pone.0170828.s001.docx]

**Additional file 1 - Database-specific search terms and strategies**

**Search strategy for MEDLINE (conducted December, 5, 2016)**

**#1 Preterm**

"Infant, Premature"[Mesh] OR "Premature Birth"[Mesh] OR "Infant, Very Low Birth Weight"[Mesh] OR prematur*[tiab] OR preterm[tiab] OR pre-mature[tiab] OR pre-term[tiab] OR "Low Birth Weight"[tiab] OR VLBW[tiab] OR LBW[tiab] OR ELBW[tiab]

**#2 Sensory functioning**

"Sensation"[Mesh] OR "sensory modulation"[tiab] OR "sensory processing"[tiab] OR "sensory functioning"[tiab] OR "sensory responsiveness"[tiab] OR "sensory profile"[tiab] OR "sensory profiles"[tiab] OR “sensory reactivity”[tiab]

**#3 Questionnaire/ test**

"Surveys and Questionnaires"[Mesh] OR Questionnair*[tiab] OR rating[tiab] OR scale[tiab] OR test[tiab] OR tests[tiab] OR "sensory testing"[tiab] OR "Sensory Profile"[tiab] OR "test of sensory functions in infants"[tiab] OR TSFI[tiab] OR SRS[tiab] OR SP[tiab] OR "infant toddler sensory profile"[tiab] OR ITSP[tiab] OR SSP[tiab]

**#4 Diagnosis**

"sensory processing disorder"[tiab] OR SPD[tiab] OR "sensory modulation disorder"[tiab] OR SMD[tiab]

**#5 Publicatie type filter**

NOT ("addresses"[Publication Type] OR "biography"[Publication Type] OR "comment"[Publication Type] OR "directory"[Publication Type] OR "editorial"[Publication Type] OR "festschrift"[Publication Type] OR "interview"[Publication Type] OR "lectures"[Publication Type] OR "legal cases"[Publication Type] OR "legislation"[Publication Type] OR "letter"[Publication Type] OR "news"[Publication Type] OR "newspaper article"[Publication Type] OR "patient education handout"[Publication Type] OR "popular works"[Publication Type] OR "congresses"[Publication Type] OR "consensus development conference"[Publication Type] OR "consensus development conference, nih"[Publication Type] OR "practice guideline"[Publication Type] OR "Dissertations, Academic as Topic"[Mesh]) NOT (animals[mh] NOT humans[mh])

**(#1 AND #2 AND (#3 OR #4)) NOT #5**

| **Search** | **PubMed Query 05-12-2016** | **Items found** |
| --- | --- | --- |
| [#5](https://www.ncbi.nlm.nih.gov/pubmed)#5 | Search **#1 AND #2 AND (#3 OR #4)** | [507](https://www.ncbi.nlm.nih.gov/pubmed/?cmd=HistorySearch&querykey=5) |
| [#4](https://www.ncbi.nlm.nih.gov/pubmed)#4 | Search **"sensory processing disorder"[tiab] OR SPD[tiab] OR "sensory modulation disorder"[tiab] OR SMD[tiab]** | [6260](https://www.ncbi.nlm.nih.gov/pubmed/?cmd=HistorySearch&querykey=4) |
| [#3](https://www.ncbi.nlm.nih.gov/pubmed)#3 | Search **"Surveys and Questionnaires"[Mesh] OR Questionnair*[tiab] OR rating[tiab] OR scale[tiab] OR test[tiab] OR tests[tiab] OR "sensory testing"[tiab] OR "Sensory Profile"[tiab] OR "test of sensory functions in infants"[tiab] OR TSFI[tiab] OR SRS[tiab] OR SP[tiab] OR "infant toddler sensory profile"[tiab] OR ITSP[tiab] OR SSP[tiab]** | [2893243](https://www.ncbi.nlm.nih.gov/pubmed/?cmd=HistorySearch&querykey=3) |
| [#2](https://www.ncbi.nlm.nih.gov/pubmed)#2 | Search **"Sensation"[Mesh] OR "sensory modulation"[tiab] OR "sensory processing"[tiab] OR "sensory functioning"[tiab] OR "sensory responsiveness"[tiab] OR "sensory profile"[tiab] OR "sensory profiles"[tiab] OR "sensory reactivity"[tiab]** | [268127](https://www.ncbi.nlm.nih.gov/pubmed/?cmd=HistorySearch&querykey=2) |
| [#1](https://www.ncbi.nlm.nih.gov/pubmed)#1 | Search **"Infant, Premature"[Mesh] OR "Premature Birth"[Mesh] OR "Infant, Very Low Birth Weight"[Mesh] OR prematur*[tiab] OR preterm[tiab] OR pre-mature[tiab] OR pre-term[tiab] OR "Low Birth Weight"[tiab] OR VLBW[tiab] OR LBW[tiab] OR ELBW[tiab]** | [191310](https://www.ncbi.nlm.nih.gov/pubmed/?cmd=HistorySearch&querykey=1) |

**Search strategy for EMBASE (conducted December, 5, 2016)**

**#1 Preterm**

'prematurity'/exp OR 'very low birth weight'/exp OR prematur*:ti,ab OR preterm:ti,ab OR pre-mature:ti,ab OR pre-term:ti,ab OR VLBW:ti,ab OR "Low Birth Weight":ti,ab OR LBW:ti,ab OR ELBW:ti,ab

**#2 Sensory functioning**

'sensation'/de OR 'abnormal sensation'/exp OR 'sensory dysfunction'/de OR 'sensory modulation':ti,ab OR 'sensory processing':ti,ab OR 'sensory functioning':ti,ab OR 'sensory responsiveness':ti,ab OR 'sensory profile':ti,ab OR 'sensory profiles':ti,ab OR 'sensory reactivity':ti,ab

**#3 Questionnaire/ test**

'questionnaire'/exp OR Questionnair*:ti,ab OR rating:ti,ab OR scale:ti,ab OR test:ti,ab OR tests:ti,ab OR 'sensory testing':ti,ab OR 'Sensory Profile':ti,ab OR 'test of sensory functions in infants':ti,ab OR TSFI:ti,ab OR SRS:ti,ab OR SP:ti,ab OR 'infant toddler sensory profile':ti,ab OR ITSP:ti,ab OR SSP:ti,ab

**#4 Diagnosis**

'sensory processing disorder':ti,ab OR SPD:ti,ab OR 'sensory modulation disorder':ti,ab OR SMD:ti,ab

**#5 Limits: Publication type & humans:**

([article]/lim OR [article in press]/lim OR [review]/lim OR [short survey]/lim) AND [humans]/lim

## Embase Session Results (5 Dec 2016)

| No. | Query | Results |
| --- | --- | --- |
| #6 | **#5** AND ([article]/lim OR [article in press]/lim OR [review]/lim OR [short survey]/lim) AND [humans]/lim | **59** |
| #5 | **#1** AND **#2** AND (**#3** OR **#4**) | **101** |
| #4 | **'sensory processing disorder'**:ti,ab OR **spd**:ti,ab OR **'sensory modulation disorder'**:ti,ab OR **smd**:ti,ab | **7667** |
| #3 | **'questionnaire'**/exp OR **questionnair***:ti,ab OR **rating**:ti,ab OR **scale**:ti,ab OR **test**:ti,ab OR **tests**:ti,ab OR **'sensory testing'**:ti,ab OR **'sensory profile'**:ti,ab OR **'test of sensory functions in infants'**:ti,ab OR **tsfi**:ti,ab OR **srs**:ti,ab OR **sp**:ti,ab OR **'infant toddler sensory profile'**:ti,ab OR **itsp**:ti,ab OR **ssp**:ti,ab | **3249674** |
| #2 | **'sensation'**/de OR **'abnormal sensation'**/exp OR **'sensory dysfunction'**/de OR **'sensory modulation'**:ti,ab OR **'sensory processing'**:ti,ab OR **'sensory functioning'**:ti,ab OR **'sensory responsiveness'**:ti,ab OR **'sensory profile'**:ti,ab OR **'sensory profiles'**:ti,ab OR **'sensory reactivity'**:ti,ab | **37743** |
| #1 | **'prematurity'**/exp OR **'very low birth weight'**/exp OR **prematur***:ti,ab OR **preterm**:ti,ab OR **'pre mature'**:ti,ab OR **'pre term'**:ti,ab OR **vlbw**:ti,ab OR **'low birth weight'**:ti,ab OR **lbw**:ti,ab OR **elbw**:ti,ab | **249648** |

**Search strategy for PsycINFO (conducted December, 5, 2016)**

**#1 Preterms**

DE "Premature Birth" OR DE "Premature Birth" OR TI prematur* OR TI "Very Low Birth Weight" OR TI preterm OR TI pre-mature OR TI pre-term OR TI VLBW OR TI "Low Birth Weight" OR TI LBW OR TI ELBW OR AB prematur* OR AB "Very Low Birth Weight" OR AB preterm OR AB pre-mature OR AB pre-term OR AB VLBW OR AB "Low Birth Weight" OR AB LBW OR AB ELBW

**#2 Sensory functioning**

TI "sensory modulation" OR TI "sensory processing" OR TI "sensory functioning" OR TI "sensory responsiveness" OR TI "sensory profile" OR TI "sensory profiles" OR TI sensory reactivity OR AB "sensory modulation" OR AB "sensory processing" OR AB "sensory functioning" OR AB "sensory responsiveness" OR AB "sensory profile" OR AB "sensory profiles" OR AB sensory reactivity

**#3 Questionnaire/ test**

DE "Sensorimotor Measures" OR DE "Questionnaires" OR DE "Surveys" OR DE "Testing" OR DE "Rating" OR DE "Rating Scales" OR TI Questionnair* OR TI rating OR TI scale OR TI test OR TI tests OR TI "sensory testing" OR TI "Sensory Profile" OR TI "test of sensory functions in infants" OR TI TSFI OR TI SRS OR TI "infant toddler sensory profile" OR TI ITSP OR TI SSP OR AB Questionnair* OR AB rating OR AB scale OR AB test OR AB tests OR AB "sensory testing" OR AB "Sensory Profile" OR AB "test of sensory functions in infants" OR AB TSFI OR AB SRS OR AB "infant toddler sensory profile" OR AB ITSP OR AB SSP

**#4 Diagnosis**

TI "sensory processing disorder" OR TI SPD OR TI "sensory modulation disorder" OR TI SMD OR AB "sensory processing disorder" OR AB SPD OR AB "sensory modulation disorder" OR AB SMD

**# Publication type**

Bottom of Form

| **#** | **PsycINFO Query 05-12-2016** | **Limiters/Expanders** | **Results** |
| --- | --- | --- | --- |
| S5 | S1 AND S2 AND (S3 OR S4) | Limiters - Publication Type: All Journals, Peer Reviewed Journal, Peer-Reviewed Status-Unknown; Population Group: Human; Document Type: Abstract Collection, Journal Article  Search modes - Boolean/Phrase | 15 |
| S4 | TI "sensory processing disorder" OR TI SPD OR TI "sensory modulation disorder" OR TI SMD OR AB "sensory processing disorder" OR AB SPD OR AB "sensory modulation disorder" OR AB SMD | Search modes - Boolean/Phrase | 1,324 |
| S3 | DE "Sensorimotor Measures" OR DE "Questionnaires" OR DE "Surveys" OR DE "Testing" OR DE "Rating" OR DE "Rating Scales" OR TI Questionnair* OR TI rating OR TI scale OR TI test OR TI tests OR TI "sensory testing" OR TI "Sensory Profile" OR TI "test of sensory functions in infants" OR TI TSFI OR TI SRS OR TI "infant toddler sensory profile" OR TI ITSP OR TI SSP OR AB Questionnair* OR AB rating OR AB scale OR AB test OR AB tests OR AB "sensory testing" OR AB "Sensory Profile" OR AB "test of sensory functions in infants" OR AB TSFI OR AB SRS OR AB "infant toddler sensory profile" OR AB ITSP OR AB SSP | Search modes - Boolean/Phrase | 902,972 |
| S2 | TI "sensory modulation" OR TI "sensory processing" OR TI "sensory functioning" OR TI "sensory responsiveness" OR TI "sensory profile" OR TI "sensory profiles" OR TI sensory reactivity OR AB "sensory modulation" OR AB "sensory processing" OR AB "sensory functioning" OR AB "sensory responsiveness" OR AB "sensory profile" OR AB "sensory profiles" OR AB sensory reactivity | Search modes - Boolean/Phrase | 3,244 |
| S1 | DE "Premature Birth" OR DE "Premature Birth" OR TI prematur* OR TI "Very Low Birth Weight" OR TI preterm OR TI pre-mature OR TI pre-term OR TI VLBW OR TI "Low Birth Weight" OR TI LBW OR TI ELBW OR AB prematur* OR AB "Very Low Birth Weight" OR AB preterm OR AB pre-mature OR AB pre-term OR AB VLBW OR AB "Low Birth Weight" OR AB LBW OR AB ELBW | Search modes - Boolean/Phrase | 20,134 |
