# Supplementary material for: Sensory modulation in preterm children: Theoretical perspective and systematic review
Source: PLoS One. 2017 Feb 9;12(2):e0170828. doi: 10.1371/journal.pone.0170828 (PMC5300179; doi:10.1371/journal.pone.0170828)
Supplement: S3 File — (DOCX) [file pone.0170828.s003.docx]

**Supplemental file S3 - Data Extraction Form**

- Reference (first author /year of publication/journal)
- Sample size:

Preterm/intervention sample (#)

Control sample (#)

- Definition of prematurity:
  - General = < 37 weeks gestation
  - Moderate preterm = 32-37 weeks gestation
  - Very preterm = 28-32 weeks gestation
  - extremely preterm= < 28 weeks gestation
- Age at assessment (months/ years)
  - Corrected age (YES/NO)
- Main outcome measures:
  - Sensory functioning:
    - Standardized test (write-in)
    - Caregiver questionnaire (write-in)
    - Clinical exam (write-in)
  - Perinatal risk factors (write-in)
  - Behavioral functioning:
    - Caregiver questionnaire (write-in)
  - Neurocognitive functioning:
    - Standardized test (write-in)
    - Caregiver questionnaire (write-in)
- Study design:
  - Cross-sectional
  - Intervention/RCT
  - Population-based
  - Other
- Continent/country
- Quality score (Newcastle-Ottowa Scale):
  - Additional: Norm referenced group? (YES/NO)
- Risk of Bias analysis
  - Objectives adequately described (YES/NO)
  - Characteristics of participants adequately described (YES/NO)
  - Group selection adequately described (YES/NO)
  - Exclusion/inclusion criteria defined (YES/NO)
  - Matching of subjects (YES/NO + write-in)
  - Attrition described (YES/NO)
  - Conflict of interest described (YES/NO)
  - Performance bias described (YES/NO)
  - Reporting bias (YES/NO)
- Key results:
  - Sensory modulation (% and/or p-values)
  - Perinatal risk factors (R, R^2^, r, OR, t -values)
  - Behavioral/neurocognitive measures (R, R^2^, r, OR, t -values)
- Notes (write-in)
